# Variants of Erythema Multiforme: A Case Report and Literature Review

**DOI:** 10.7759/cureus.3459

**Published:** 2018-10-16

**Authors:** Luis Paulino, David J Hamblin, Ngozi Osondu, Richard Amini

**Affiliations:** 1 Medical Education and Simulation, University of Arizona College of Medicine, Tucson, USA; 2 Infectious Diseases, University of Arizona College of Medicine, Tucson, USA; 3 Emergency Medicine, University of Arizona College of Medicine, Tucson, USA

**Keywords:** erythema multiform, erythema multiform major, erythema multiform minor, steven-johnson syndrome

## Abstract

Erythema multiforme is an acute skin condition characterized by targetoid lesions and occurs most frequently in young adults, particularly males. There are two variants of this condition, one with mucosal involvement, termed erythema multiforme major, and one without mucosal involvement, known as erythema multiforme minor. Due to the similarities in clinical and histological findings, it was previously believed that erythema multiforme major was indistinguishable from Steven-Johnson syndrome (SJS). However, evidence suggests these are two distinct diseases with a different etiology. It is important for clinicians to readily identify the difference between erythema multiforme from SJS, as the prognosis and mortality rate vary significantly between the two disorders.

## Introduction

Erythema multiforme has been associated with multiple etiologies, including medications, malignancies, and sarcoidosis, but about 90% of the cases can be attributed to infectious agents, more commonly herpes simplex virus in adults and mycoplasma pneumonia in children [1]. About 10% of the cases, the symptoms are associated with an adverse drug reaction, usually to non-steroidal anti-inflammatory drugs, sulfonamides, anti-epileptics, or antibiotics [1-2]. A herpes simplex virus infection can cause the release of IFN-gamma and the subsequent recruitment of CD4+ T helper cells, which may lead to epidermal tissue damage and the pathological findings associated with erythema multiforme [2]. In drug-induced erythema multiforme, TNF-alpha has been demonstrated to cause the development of the lesions [2].

## Case presentation

A 23-year-old Hispanic male presented to the emergency department, with rash, mouth sores, and subjective fevers that began after eating fish five days prior. His symptoms started with sores in his mouth and on his lips with penile and anal pruritus. After 24 hours, the patient developed a pruritic rash over his upper extremities, neck, upper back, and palms, as well as two non-painful sores on his penis and one blister on his rectum. Despite medicating at home with Benadryl, the patient’s symptoms persisted, which caused the patient to seek care in our emergency department.

**Figure 1:**
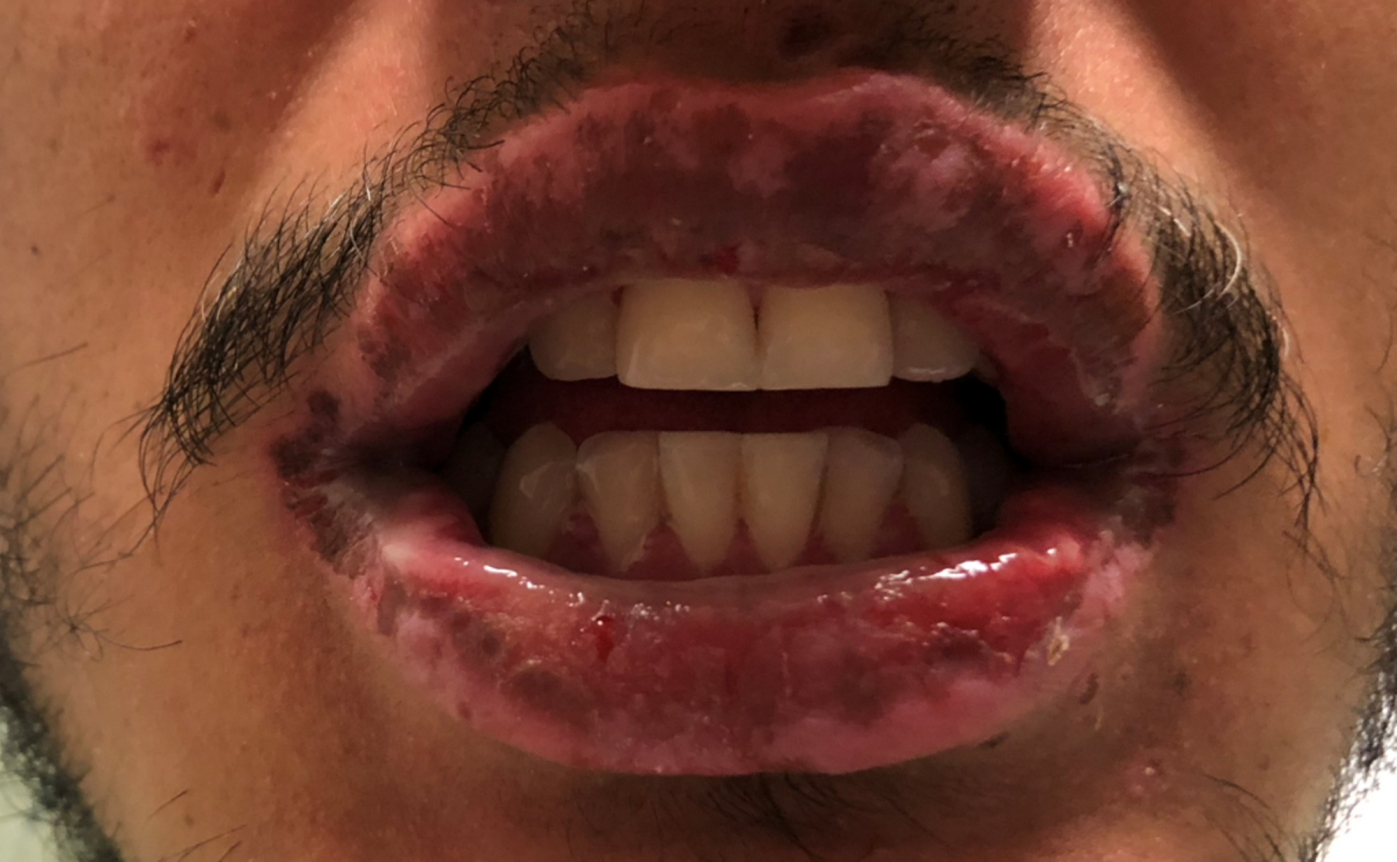
Oral Mucosa

**Figure 2:**
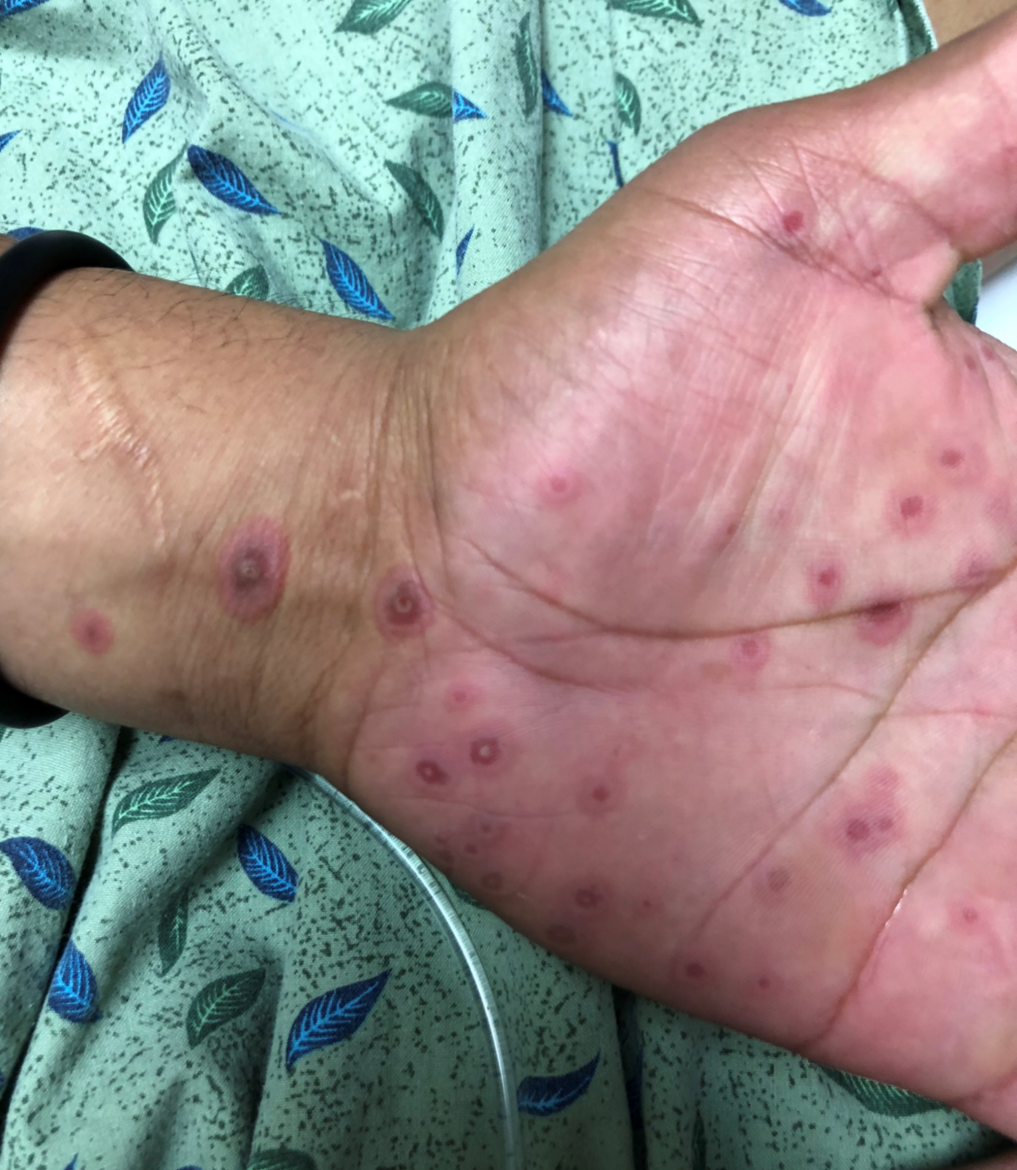
Palm Lesions

**Figure 3:**
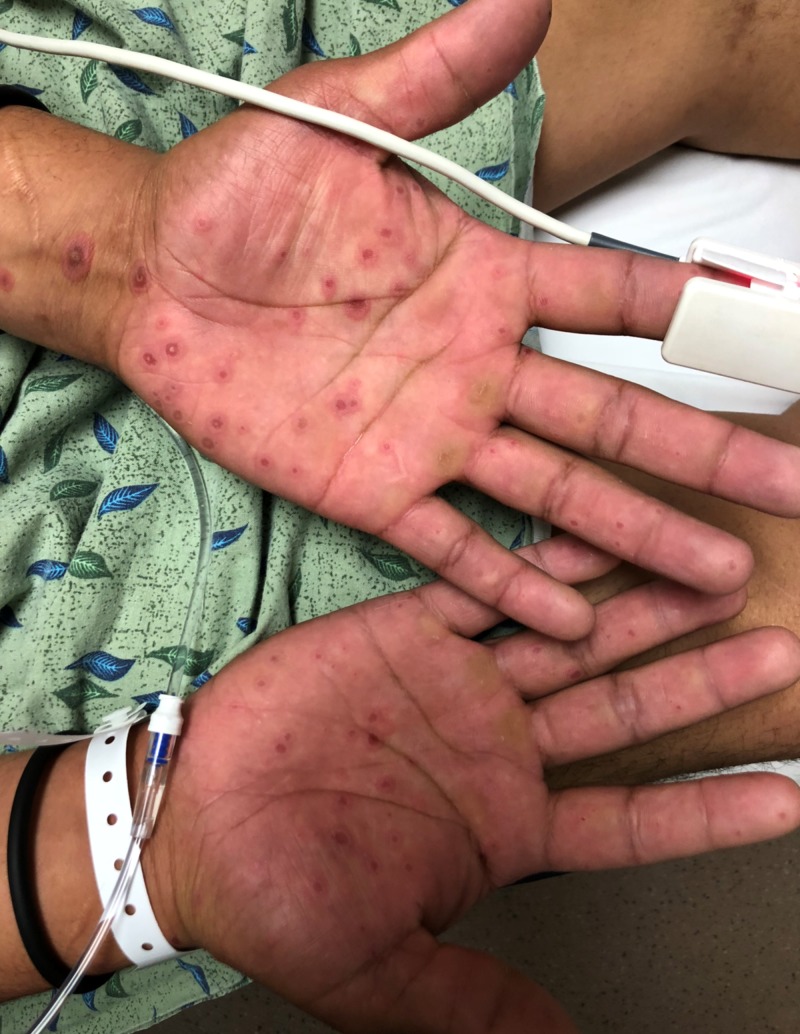
Palm Lesions Bilateral

**Figure 4:**
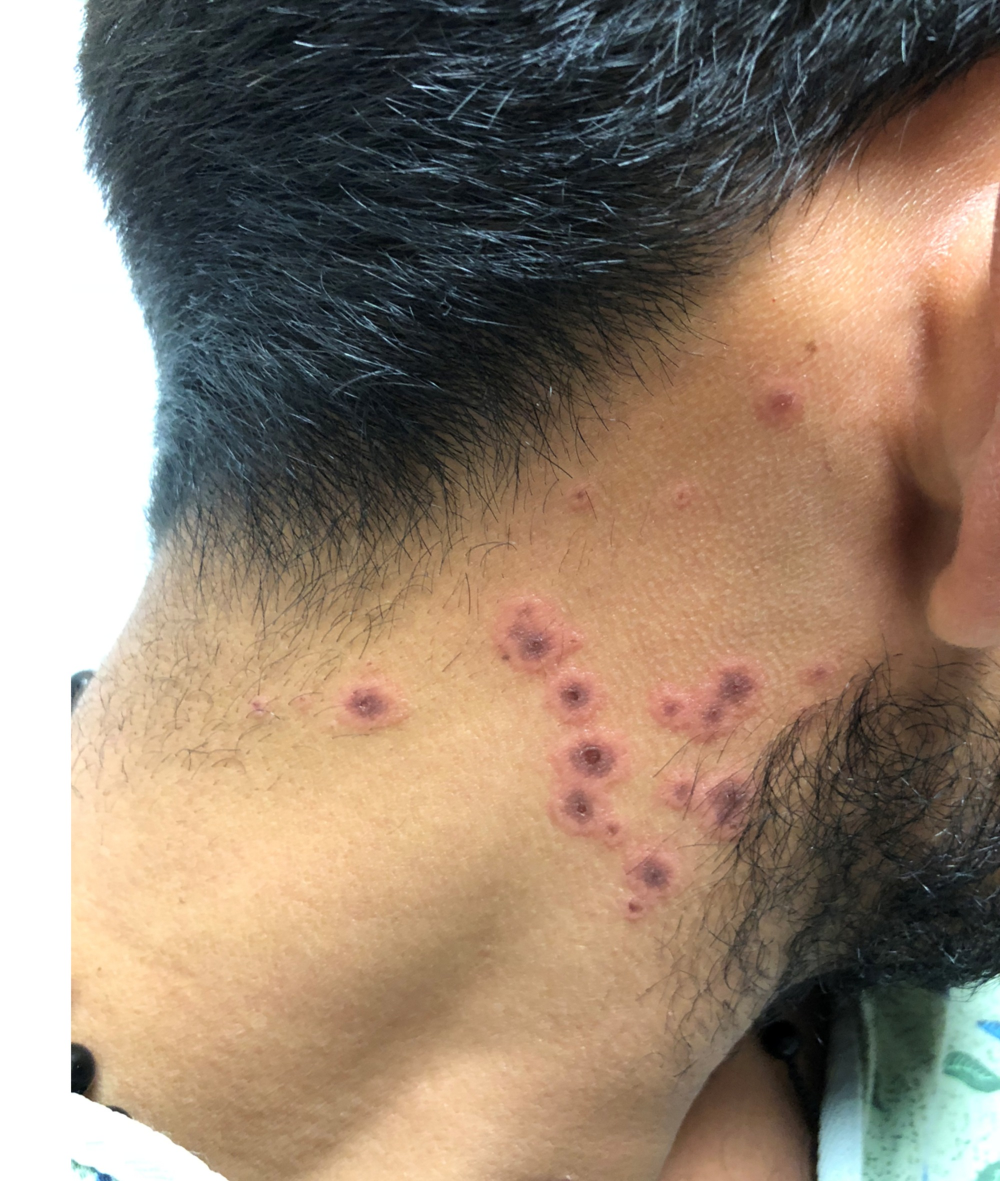
Lateral Neck

At presentation, the patient was alert and calm, without anxiety or an ill appearance. The patient reported having unprotected intercourse with a female two months ago. He denied ever having anal intercourse, a history of sexually transmitted infections, dysuria, or penile discharge. He also denied any past medical problems and did not take any prescription medications or over-the-counter supplements. The patient’s vitals were within reference range. On physical examination, he had heme-crusted polycyclic erosions of vermillion lips, buccal mucosa, and labial mucosa (Figure 1). He was also found to have numerous 2-12 mm erythematous, urticarial, targetoid papules and plaques with central hyperpigmented purple/red duskiness over bilateral palms (Figure 2, Figure 3), dorsal hands, upper arms, lateral neck (Figure 4), cheeks, nasal tip, and alae. He had several urticarial, targetoid papules with central duskiness over the penile shaft. Cardiovascular, neurologic, respiratory, and abdominal examinations were otherwise unremarkable. Both dermatology and infectious disease were consulted on this patient.

Laboratory work for this patient consisted of complete blood count, comprehensive metabolic panel, sexually transmitted infection testing, bacterial and viral blood cultures and serology, viral direct detection test, and anti-nuclear antibody testing. Of note, the patient had a white blood cell count of 5.4x10^9/L, hemoglobin of 14.7 gm/dL, platelet count of 302x10^9/L, and creatinine of 0.85 mg/dL. The patient was negative for herpes simplex virus (HSV) and human immunodeficiency virus (HIV) while positive for anti-nuclear antibody (ANA). At the time of discharge, the only test the patient was found to be positive for was anti-nuclear antibodies. He was initially treated with acyclovir, but the medication was discontinued after negative laboratory testing. Biopsy of a lesion on the patient’s upper arm exhibited interface dermatitis, consistent with erythema multiform. The patient was treated with “magic mouthwash,” consisting of Benadryl, Maalox, and lidocaine, and instructed to continue with the treatment as symptoms persisted. On the day of discharge, the patient’s rash and sores were improving and he did not have any new lesions.

## Discussion

Erythema multiforme major, minor, and persistent variations

Clinically, erythema multiforme can be categorized into major, minor, and persistent variations. The condition can present with either typical or atypical skin lesions. Targetoid lesions located on the extensor surfaces of the acral extremities are the hallmark presentation for this disorder [3]. These lesions are composed of a dusky central blister, a dark red inflammatory zone surrounded by a pale ring of edema, and an erythematous halo on the periphery of the lesion [4]. The atypical lesions appear as a raised, edematous lesion with two zones of color change and a poorly defined border [1]. Involvement of the face, neck, palms, soles, flexor surfaces, and trunk may occur with typical or atypical presentations. Lesions may also manifest in the mucous membranes of the oral, ocular, or genital mucosa and can occur with or without the associated cutaneous lesions [5]. Oral involvement is estimated to occur with 25%-60% of patients with erythema multiforme [2]. The severity of disease and the location of the lesions help to further differentiate erythema multiforme into its major and minor forms. While both variants share many of the same characteristics, erythema multiforme major is associated with a more severe presentation and is identified by lesions involving one or more mucosal membranes [2]. In contrast, erythema multiforme minor often presents with minimal to no mucosal membrane involvement and milder cutaneous symptoms [2]. There are no laboratory tests that can aid in the diagnosis of erythema multiforme when it is suspected in a patient; however, if autoimmune blistering disorders are in the differential diagnosis, serologic testing for autoantibodies such as antinuclear antibodies should be considered [6].

Lesions associated with erythema multiforme typically appear over the course of three to five days and resolve within one to two weeks. However, the more severe cases of erythema multiforme with the involvement of the mucous membranes may take up to six weeks to resolve [2]. These episodes may recur on an average of six times per year, lasting up to six to 10 years. This subset of the disorder is known as recurrent erythema multiforme and may be associated with a herpes simplex virus infection [2]. In rare instances, there is a continuous appearance of lesions without interruption that may continue for longer than one year. This variant is known as persistent erythema multiforme and may be associated with viral infections [2].

The clinical course of erythema multiforme is self-limited; however, intervention with corticosteroids can provide patients with mild symptoms some relief. Patients with severe mucosal involvement and pain reported improvements with strong systemic glucocorticoids [2]. In patients with recurrent erythema multiforme, antiviral therapy has shown to be an effective prophylaxis. The best response to therapy occurs in patients with a clear correlation between herpes simplex infection and erythema multiforme [2].

Steven-Johnson/toxic epidermal necrolysis

Steven-Johnson syndrome (SJS) and toxic epidermal necrolysis (TEN) are classified as a spectrum of diseases, distinguished solely by severity [7]. This disorder is triggered by medications and causes a severe mucocutaneous reaction that leads to epidermal detachment and necrosis [8]. SJS is the less severe disorder and is identified when there is less than 10% skin involvement. When skin involvement is over 30%, the condition is then classified as TEN [9]. In approximately 87%-100% of the cases, mucous membranes are involved, including ocular, oral, and genital [10]. SJS/TEN can occur in patients of any age and is more commonly found in women than in men. It is a relatively rare disorder with an estimated incidence rate of 5.76 cases per million people per year [11]. The overall mortality rate ranges approximately from 23% at six weeks and up to 34% at one year [12]. The majority of cases are caused by an adverse reaction to a medication; however, up to 25% of cases cannot be attributed to drugs. The next most common trigger is due to infection by Mycoplasma pneumoniae, especially in children [13].

The clinical presentation of SJS/TEN often includes fever, skin tenderness, blistering, exanthematous eruption, and mucositis. Skin lesions appear as ill-defined, erythematous macules with purpuric centers or can also appear as diffuse erythema. Ocular involvement is commonly observed and can range from acute conjunctivitis with purulent discharge and eyelid edema to corneal erosion and ulceration [10]. The involvement of the scalp, palms, and soles is typically not observed. Lesions will progress to vesicles and bullae and begin extensive shedding of skin within days [3].

Once a diagnosis of SJS/TEN has been made, physicians should determine the severity and prognosis of the disease soon after. This can be accomplished by applying a prognostic scoring system known as score of toxic epidermal necrosis (SCORTEN). Patients with limited skin involvement and a SCORTEN score of zero to one can be treated in non-specialized wards. Patients with a score greater than two should be transferred to intensive care units or burn units if available [4]. Treatment involves the withdrawal of the culprit drug and supportive wound care involving fluids, nutrition, pain control and prevention, and treatment of infection. It is important to note that prophylactic systemic antibiotics are not advised but rather the use of antiseptic solutions for disinfection is recommended [14].

## Conclusions

In the past, it was thought that erythema multiforme belonged as a part of the SJS/TEN spectrum of diseases, most likely due to the similar clinical presentation of these disorders. However, there is strong evidence to suggest that erythema multiforme is a distinct and separate condition and should not be associated with SJS/TEN. It is important to differentiate between these conditions due to the varying etiology, treatment, and prognosis of each disorder.
